# The effect of centralized care on the management of postoperative fluctuations in plasma sodium concentration after pediatric suprasellar brain tumor surgery

**DOI:** 10.1007/s11102-026-01666-w

**Published:** 2026-03-29

**Authors:** S. C. Hulsmann, D. C. Zaal, J.P.J. van Gestel, M. Sie, O.H.J. Eelkman Rooda, K.M. van Baarsen, R.E.A. Musson, B. Bakker, E.E.S. Nieuwenhuis, E.W. Hoving, R.M. Wösten-van Asperen, H. M. van Santen

**Affiliations:** 1 Princess Máxima Center for Pediatric Oncology, Heidelberglaan 25, Utrecht, 3584 CS The Netherlands; 2 Department of Pediatric Endocrinology, Wilhelmina Children’s Hospital, University Medical Center Utrecht, Lundlaan 6, Utrecht, 3584 EA The Netherlands; 3 Department of Pediatric Intensive Care, Wilhelmina Children’s Hospital, University Medical Center Utrecht, Lundlaan 6, Utrecht, 3584 EA The Netherlands; 4 Department of Clinical Chemistry, University Medical Center Utrecht, Lundlaan 6, Utrecht, 3584 EA The Netherlands

**Keywords:** Arginine vasopressin deficiency, Diabetes insipidus, Plasma sodium concentration, Suprasellar tumor, Pediatric, Centralized care

## Abstract

**Purpose:**

Children undergoing neurosurgery for (supra)sellar tumors are at risk of developing arginine vasopressin-deficiency (AVP-D), which can cause severe sodium fluctuations and associated neurological complications, prolonged hospitalization and mortality. A previous Dutch study reported sodium shifts ≥ 10 mmol/L/24 h in 75.3% of patients with early postoperative AVP-D, with a maximum delta of 46 mmol/L/24 h. Since 2018, pediatric oncology care has been centralized in the Netherlands. We evaluated the impact of this centralization on postoperative sodium fluctuations in children with (supra)sellar tumors.

**Methods:**

Data from all children who underwent neurosurgery for a (supra)sellar tumor at the Princess Máxima Centrum (Utrecht, the Netherlands) between January 2018 and December 2023 were retrospectively collected from electronic health records, including presence of preoperative AVP-D, plasma sodium concentrations during the first 10 postoperative days, administration of desmopressin, and short- and long-term neurological symptoms. Results were additionally interpreted in the context of pre-centralization data.

**Results:**

Among 73 patients with a median age of 7.9 years (range 0-17.5) at tumor resection, postoperative AVP-D occurred in 69.7%. During the first 10 postoperative days, sodium fluctuations ≥ 10 mmol/L/24 h were seen in 49.1% of these patients, with a maximum delta of 30 mmol/L/24 h. Since 2018, sodium fluctuations diminished, with a maximum delta plasma sodium of 14 mmol/L/24 h in 2023. No association was found between early postoperative AVP-D and occurrence of neurological symptoms.

**Conclusion:**

Trends observed after centralization of care for children with (supra)sellar tumors may suggest improved postoperative AVP-D management, resulting in less frequent and less severe fluctuations in plasma sodium concentrations.

**Supplementary Information:**

The online version contains supplementary material available at 10.1007/s11102-026-01666-w.

## Introduction

Sellar or suprasellar tumors and neurosurgical interventions in this region are strongly associated with disruption of the hypothalamic-pituitary function [1–4]. In the direct postoperative period, especially the management of arginine vasopressin-deficiency (AVP-D), formerly referred to as diabetes insipidus (DI) [5], can be challenging, due to occurrence of the so-called triphasic response. This response is characterized by an initial polyuric phase due to AVP-D, followed by transient syndrome of inappropriate ADH secretion (SIADH), culminating in permanent AVP-D once AVP storages are depleted [6, 7]. Prompt recognition and careful monitoring are essential to prevent severe fluctuations in plasma sodium concentrations during these first postoperative days and to optimize patient outcomes. The pediatric brain is particularly sensitive to disturbances in plasma sodium concentrations and in the pediatric population, postoperative sodium fluctuations tend to be more pronounced and less predictable than in adults [7]. Hyponatremia, in particular, has been associated with seizures, encephalopathy, cerebral edema, prolonged hospitalization, and increased mortality [8–11]. The risk of neurological complications correlates more strongly with the rapidity of sodium shifts rather than the absolute levels, due to cerebral-adaptive mechanisms [12, 13]. While untreated hyponatremia can be life-threatening, overly rapid correction may cause abrupt shifts in plasma sodium levels, leading to water extraction from brain cells, which can result in cellular dehydration and osmotic demyelination syndrome (ODS) [14, 15]. Conversely, overly rapid correction of hypernatremia can also be dangerous, due to rapid influx of water into the brain cells, resulting in cerebral edema. Treatment protocols must therefore balance urgency with caution. It has been recommended to aim for a maximum fluctuation of plasma sodium of 10 mmol/L/24 hours [16].

In a large-scale survey amongst individuals with AVP-D, patients rated health care professionals’ (HCPs) awareness and understanding of AVP-D management as inadequate [17]. Given the complexity of managing AVP-D peri- and postoperatively and its associated fluctuations in sodium levels, institutional experience in a multidisciplinary team of HCPs plays a critical role in optimizing outcomes.

In the Netherlands, each year, approximately 25 pediatric patients are newly diagnosed with a sellar or suprasellar tumor necessitating neurosurgical intervention, with the following tumor type distribution: ~10 craniopharyngioma, ~ 10 low grade glioma and ~ 5 suprasellar germinoma [18]. Care for pediatric oncology patients (including pediatric brain tumors) has been centralized in the Netherlands since 2018 through the establishment of the Princess Máxima Center for Pediatric Oncology. Almost all newly diagnosed patients with pediatric brain tumors in the Netherlands are referred to and treated at the Princess Máxima Center. Diagnostics, pre- intra- and postoperative care for these patients is coordinated and provided within this specialized center, by a dedicated team from this hospital and the adjoined academic pediatric hospital (Wilhelmina Children’s Hospital), according to standardized multidisciplinary protocols. A multidisciplinary team, including pediatric endocrinologists, intensivists, neuro-oncologists, neurosurgeons, as well as nurses, have built expertise in managing peri- and postoperative AVP-D in children undergoing (supra)sellar tumor resection and recognize its complexity. The surgical management of pediatric (supra)sellar tumors has evolved from radical resection toward more conservative, limited resection aimed at preserving hypothalamic and pituitary integrity [19–24]. Despite this paradigm shift, a considerable proportion of patients continue to develop postoperative AVP-D [25].

Prior to centralization, postoperative fluctuations in plasma sodium levels were assessed in a national cohort of pediatric patients treated for (supra)sellar tumors in the Netherlands [26]. It was found that, despite top academic care provided by 7 centers, 75% of patients with early postoperative AVP-D having fluctuations of ≥ 10mmol/L/day. The maximum delta plasma sodium concentration in patients with early postoperative AVP-D was found to be 46 mmol/L/24 h with overall ranges of plasma sodium from 110 to 183 mmol/L. Moreover, low plasma sodium concentrations were related to short-term neurological events, such as altered mental status and the occurrence of seizures [26]. The significant postoperative sodium fluctuations in this previous cohort most likely reflect the limited clinical exposure and thus experience regarding the postoperative course of AVP-D in pediatric (supra)sellar tumor patients, which can be attributed to the relatively low incidence of these tumors in the Netherlands and their dispersion across multiple centers prior to centralization. Here, we aimed to evaluate the postoperative plasma sodium fluctuations following neurosurgical resection in children treated for (supra)sellar tumors at the Princess Máxima Center and to evaluate the impact of centralization of care on the postoperative management of AVP-D.

## Materials and methods

### Study design and population

Children who underwent neurosurgery for a sellar or suprasellar tumor since centralization of care at the Princess Máxima Centrum (Utrecht, the Netherlands) between January 2018 and December 2023, were evaluated for inclusion. Admission to the Pediatric Intensive Care Unit (PICU) following (supra)sellar tumor resection prior to transfer to the pediatric Neuro-Oncology department is standard for all patients. The pediatric neurosurgical database from the Neuro-Oncology department and the PICU database were cross-checked for duplicity, and patients were screened for eligibility. Inclusion criteria were: (1) diagnosis of a sellar or suprasellar tumor; (2) partial or gross total tumor resection performed between January 2018 and December 2023; (3) age < 18 years at first radiological diagnosis. Patients were excluded if data on plasma sodium concentration from the first three postoperative days were not available, or if patients had been diagnosed with AVP-D prior to the tumor resection (either due to tumor compression or previous tumor resection(s)). Patients with previous tumor resection(s) without AVP-D were not excluded.

### Data collection

Patient data were retrospectively retrieved from electronic health records (EHRs), including gender, age (at first radiological diagnosis, at time of resection and latest follow-up), weight, height, body mass index (BMI), presenting signs and symptoms (seizures, increased intracranial pressure, hydrocephalus, visual impairment, motor and/or cognitive deficits, preoperative AVP-D, (pan)hypopituitarism), and duration of presenting symptoms), tumor diagnosis and neurosurgical data (extent of tumor resection, neurosurgical approach), plasma sodium concentrations in mmol/L during the first ten postoperative days (highest and lowest plasma sodium concentration per day, highest and lowest plasma sodium concentration during the first ten postoperative days, delta plasma sodium concentration per 24 h, largest delta plasma sodium concentration per 24 h, total amount of fluctuations in plasma sodium concentration ≥ 10 mmol/L/24 h), postoperative course (occurrence of the triphasic response, occurrence of AVP-D, transient or permanent AVP-D, administration of desmopressin pre- and/or postoperatively, PICU length of stay), short- and long-term neurological disorders, presence of endocrine disorders at diagnosis and latest follow-up (prevalence of permanent AVP-D, (pan)hypopituitarism, (hypothalamic) obesity), adjuvant treatment given (radiotherapy, chemotherapy, and targeted therapy), and mortality.

### Definitions

AVP-D was defined as documented diagnosis of AVP-D by a (pediatric) endocrinologist in the EHR or as presence of polyuria (> 5 ml/kg/hour) in combination with a high plasma sodium concentration (> 145 mmol/L in combination with a urine osmolality < 100 mOsmol/kg). Early AVP-D was defined as the occurrence of AVP-D within ten postoperative days. AVP-D was classified as *transient* when treatment with desmopressin could be discontinued before hospital discharge. AVP-D was classified as *permanent* if desmopressin treatment could not be discontinued until last moment of follow-up. SIADH was defined as oliguria in combination with low plasma sodium concentration (< 135 mmol/L) or documentation diagnosis of SIADH by a (pediatric) endocrinologist. Early hyponatremia was defined as a first low plasma sodium concentration (< 135 mmol/L) occurring during day 1–3, delayed hyponatremia was defined as a first low plasma sodium concentration (< 135 mmol/L) occurring during day 4–10. The triphasic response was specified as a period of AVP-D in the early postoperative period, followed by a phase of SIADH, and subsequently followed by a second phase of AVP-D.

Extent of tumor resection, as reported in both surgical and radiological reports, was categorized into limited resection (LR, < 95% of tumor resected), near total resection (NTR, > 95% of tumor resected with radiologically detected tumor remnants) or gross total resection (GTR, both macroscopical and radiological complete tumor resection).

Short-term neurological sequelae possibly related to plasma sodium fluctuations were defined as epileptic seizures or altered mental status (Glasgow Coma Scale score < 15 or sleepiness/reduced alertness described in the EHRs) during the first ten postoperative days. Long-term neurological sequelae were defined as headache, visual impairment (diagnosed by ophthalmologist), motor deficit, cognitive dysfunction, sleeping problems, fatigue or epileptic seizures, as reported in EHRs. Pituitary dysfunction was defined as presence of any of the following; growth hormone (GH) deficiency, central hypothyroidism, central hypogonadism or central hypocortisolism. Panhypopituitarism was defined as presence of deficiency of three or more anterior pituitary hormones. Obesity was defined as present based on cut-off values for BMI z-scores in children [27, 28].

During the postoperative period, patients were admitted to the PICU for 48 to 72 h, where a standardized protocol (Supplement 1) was strictly followed for all cases.

### Statistical analysis

The SPSS (version 29.0.1) statistical package was used for analysis. Means and standard deviation (SD) were calculated for normal distributed variables, median and full and interquartile range (IQR) for non-normal distributed variables. Normality of the data was assessed with a QQ-plot of the residuals, Shapiro-Wilk’s test, and side-by-side boxplots for homogeneity of variances. Univariate logistic regression models were used to explore associations between tumor type and the occurrence of severe sodium fluctuations, between pituitary stalk sacrifice and the occurrence of severe sodium fluctuations, and between the occurrence of early postoperative AVP-D and short- and long-term neurological outcomes. All p values were based on two-sided testing and p values < 0.05 were considered as statistically significant.

## Results

### Patient characteristics

A total of 73 patients were included (with a female predominance of 56.2%) (Table 1). Most patients (35/73, 47.9%), had been diagnosed with a craniopharyngioma or pilocytic astrocytoma (25/73, 34.2%). Median age at tumor resection was 7.8 years (range 0–17.4.4). In 69/73 (94.5%) patients, a transcranial tumor resection was performed. Most patients (34/73, 46.6%) underwent near total tumor resection, and gross total resections were performed in 28.8% (21/73). In 24.7% (18/73) of patients limited resection was performed. Eleven out of 73 patients (15.1%) had undergone previous tumor resection without developing AVP-D. In 31/73 (42.5%) patients, (partial) anterior pituitary dysfunction was present preoperatively. The median length of stay at the PICU was 4 days (range 1–28). Median duration of follow-up after tumor resection was 2.0 years (range 0.2–5.8). Mortality rate during follow-up was 4.1% (3/73), all of which were tumor-related deaths.

**Table 1:** Patient characteristics

Total *n* (%)		73 (100)
Female *n (%)*		41 (56.2)
Tumor type *n (%)*	Craniopharyngioma	35 (47.9)
	Pilocytic astrocytoma	25 (34.2)
	Teratoma	5 (6.8)
	Other*	8 (10.9)
Year of tumor resection *n (%)*	2018	9 (12.3)
	2019	3 (4.1)
	2020	19 (26.0)
	2021	16 (21.9)
	2022	14 (19.2)
	2023	12 (16.4)
Median age at time of resection in years (range)		7.8 (0–17.4.4)
Length of stay in PICU in days (range)		4 (1–28)
Previous surgery performed *n (%)*	Tumor resection	11 (15.1)
	Cyst fenestration	5 (6.8)
	Biopsy	3 (4.1)
	Drain or shunt placement	9 (12.3)
Preoperative pituitary dysfunction *n (%)*	GH deficiency	20/73 (27.4)
	TSH deficiency	18/73 (24.7)
	ACTH deficiency	11/73 (15.1)
	Gonadotropin deficiency	4/73 (5.5)
	CPP	2/73 (2.7)
Early postoperative AVP-D *n (%)*		50 (68.5)
Transient AVP-D *n (%)*		8/50 (16.0)
Permanent AVP-D *n (%)*		42/50 (84.0)
	With triphasic response *n (%)*	23/42 (54.8)
	Without triphasic response *n (%)*	19/42 (45.2)
Radiotherapy during follow-up *n (%)*	Yes	9 (12.3)
	No	64 (87.7)
Chemotherapy during follow-up *n (%)*	Yes	30 (41.1)
	No	43 (58.9)
Median duration of follow-up in months (range)		24.0 (2–70)
Mortality rate** *n (%)*		3 (4.1)

*GH* growth hormone, *TSH* thyroid stimulating hormone, *ACTH* adrenocorticotropic hormone, *CPP* central precocious puberty, *GnRH* gonadotropin releasing hormone, *AVP-D* arginine vasopressin deficiency

*Pituitary adenoma 2/76, germinoma 3/76, rhabdomyosarcoma 1/76, non-germinoma 1/76, hamartoma 1/76, Atypical choroid plexus papilloma 1/76; **Mortality unrelated to plasma sodium fluctuations, but due to other tumor-related complications

### Frequency of postoperative fluctuations in plasma sodium concentration

Of the 73 patients, 50 (68.5%) were diagnosed with early postoperative AVP-D and received treatment with desmopressin. AVP-D was transient in 8/50 cases (16.0%) and permanent in 42/50 cases (84.0%). A triphasic response preceded the development of permanent AVP-D in 23 of these 42 patients (54.8%). The median onset of the first phase with polyuria post-resection was one day postoperatively (range 1–3 days), median onset of the second phase with oliguria was at day 5 (range 2–8 days) and median onset of the third phase (AVP-D) with polyuria was at day 9 (range 4–13). Postoperative hyponatremia occurred in 50 of 73 patients (68.5%), with early hyponatremia in 21/50 patients (42.0%) and delayed hyponatremia in 29/50 patients (58.0%).

### Range of fluctuations in plasma sodium concentration in patients with AVP-D

Fluctuations of plasma sodium concentration of ≥ 10 mmol/L/day within the first 10 postoperative days were observed in 25/50 (50%) pediatric patients with early postoperative AVP-D.

Plasma sodium fluctuations of ≥ 10 mmol/L/day within the first 10 postoperative days occurred in 16/31 (51.6%) patients with craniopharyngioma and AVP-D and in 5/11 (45.6%) patients with pilocytic astrocytoma and AVP-D. No association was found between tumor type and the occurrence of severe sodium fluctuations in the first 10 postoperative days in patients with AVP-D. Explicit reported pituitary stalk sacrifice was not found to be associated with higher occurrence of sodium fluctuations of ≥ 10 mmol/L/day within the first 10 postoperative days when assessed in all patients (*N* = 73, χ²(2) = 1.44, *p* = 0.486, Cramer’s V = 0.14) nor in the patients with early postoperative AVP-D (*N* = 50, χ²(2) = 0.324, *p* = 0.850, Cramer’s V = 0.08).

**Fig. 1:**
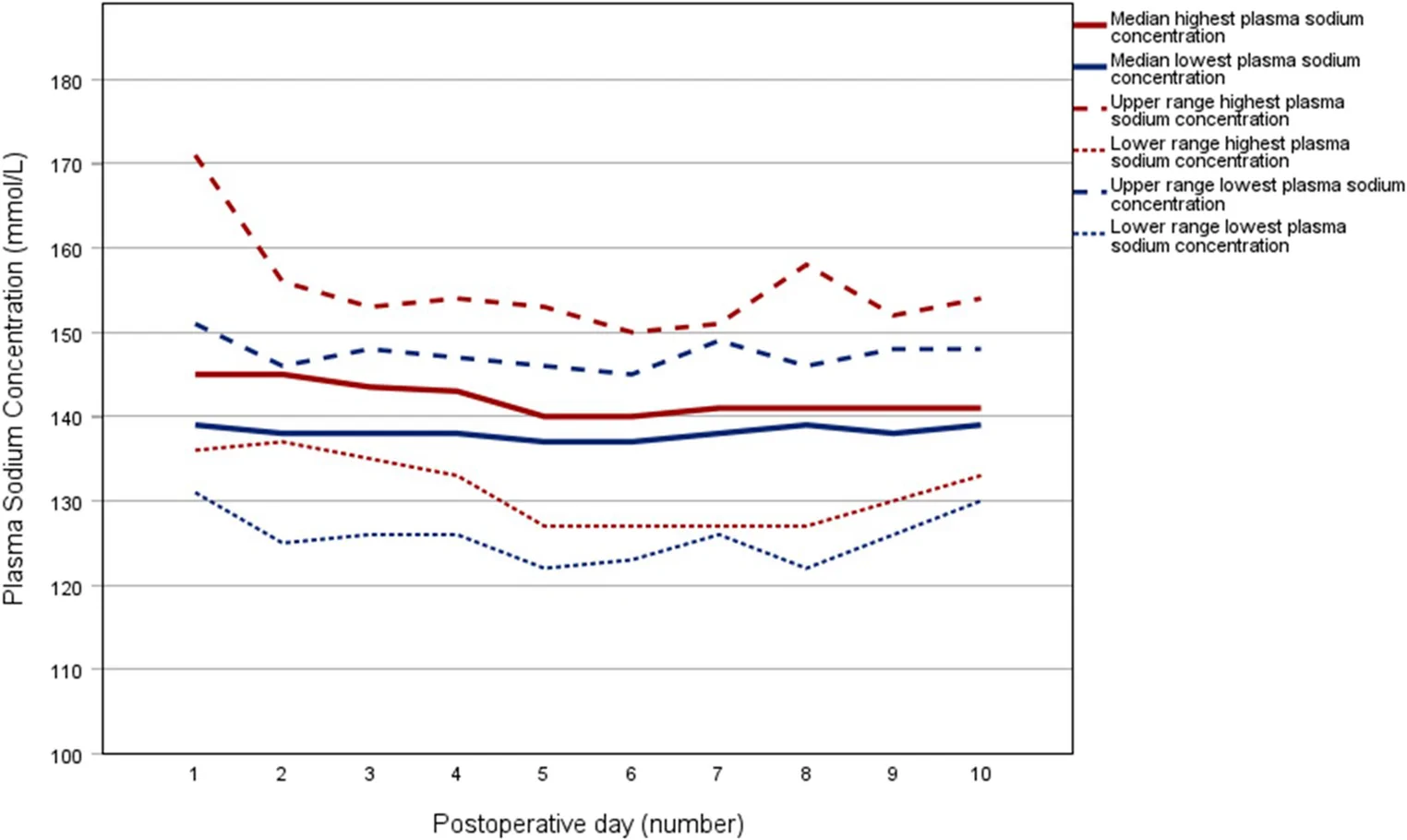
Median highest and median lowest plasma sodium concentration (mmol/L) per day (including upper and lower range) during the first 10 days after tumor surgery in patients with early postoperative AVP-D (*N* = 50) after centralization of care in the Netherlands

**Fig. 2:**
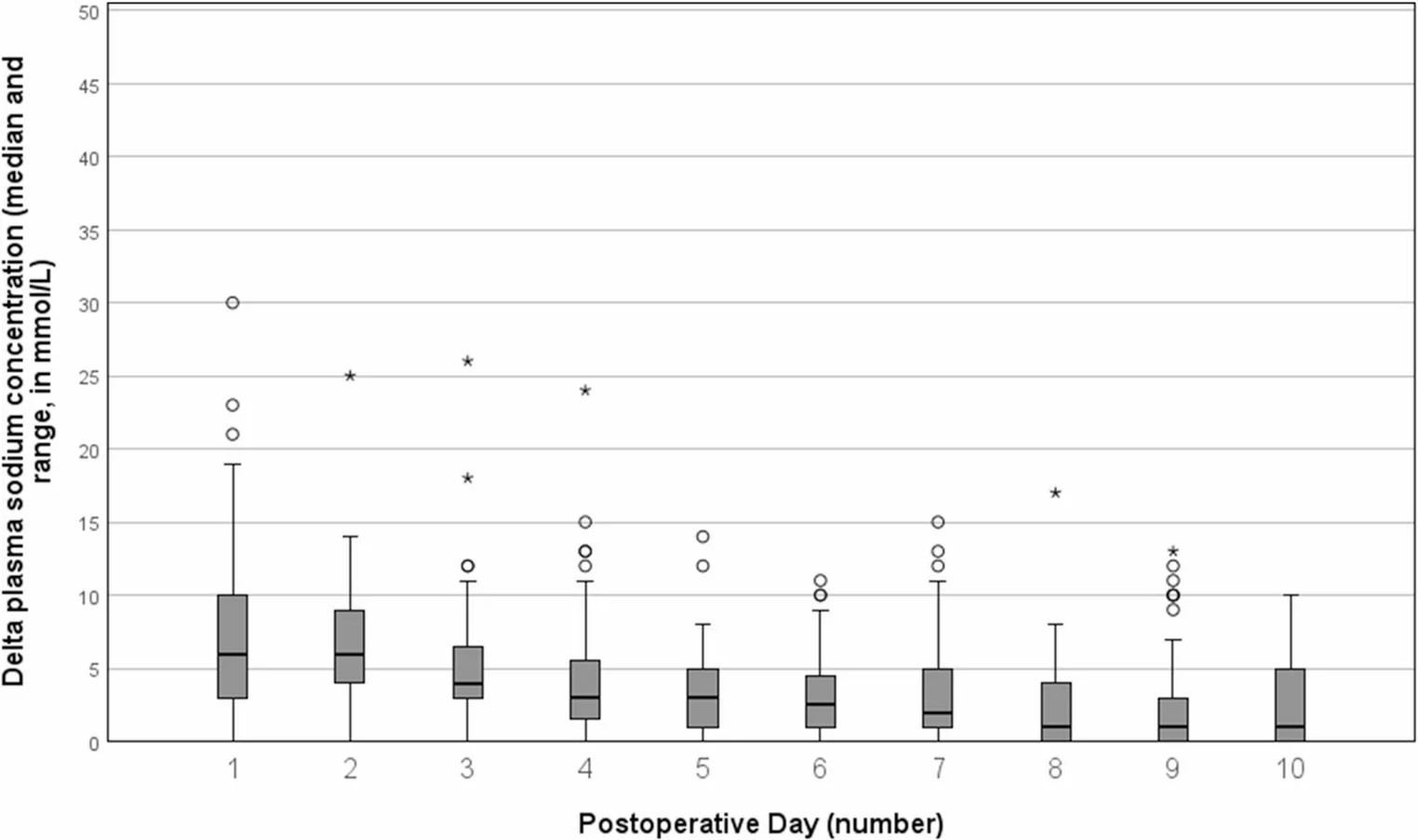
Delta plasma sodium concentration (mmol/L) per day (including median, upper and lower range) during the first 10 days after tumor surgery in patients with early postoperative AVP-D (*N* = 50) after centralization of care in the Netherlands

In Fig. 1, the course of plasma sodium concentrations during the first 10 days after tumor surgery in patients with early postoperative AVP-D (*N* = 50) is depicted. In these patients, the highest observed plasma sodium level was 171 mmol/L, which occurred on day 1 in 2018, and the lowest plasma sodium level was 122 mmol/L, which also occurred in 2018, on day 8. The largest individual delta plasma sodium concentration per day that was observed in patients with early postoperative AVP-D was 30 mmol/L and occurred on day 1 post-resection in 2018 (Fig. 2).

###  Trends in time of plasma sodium fluctuations in patients with early postoperative AVP-D

**Fig. 3:**
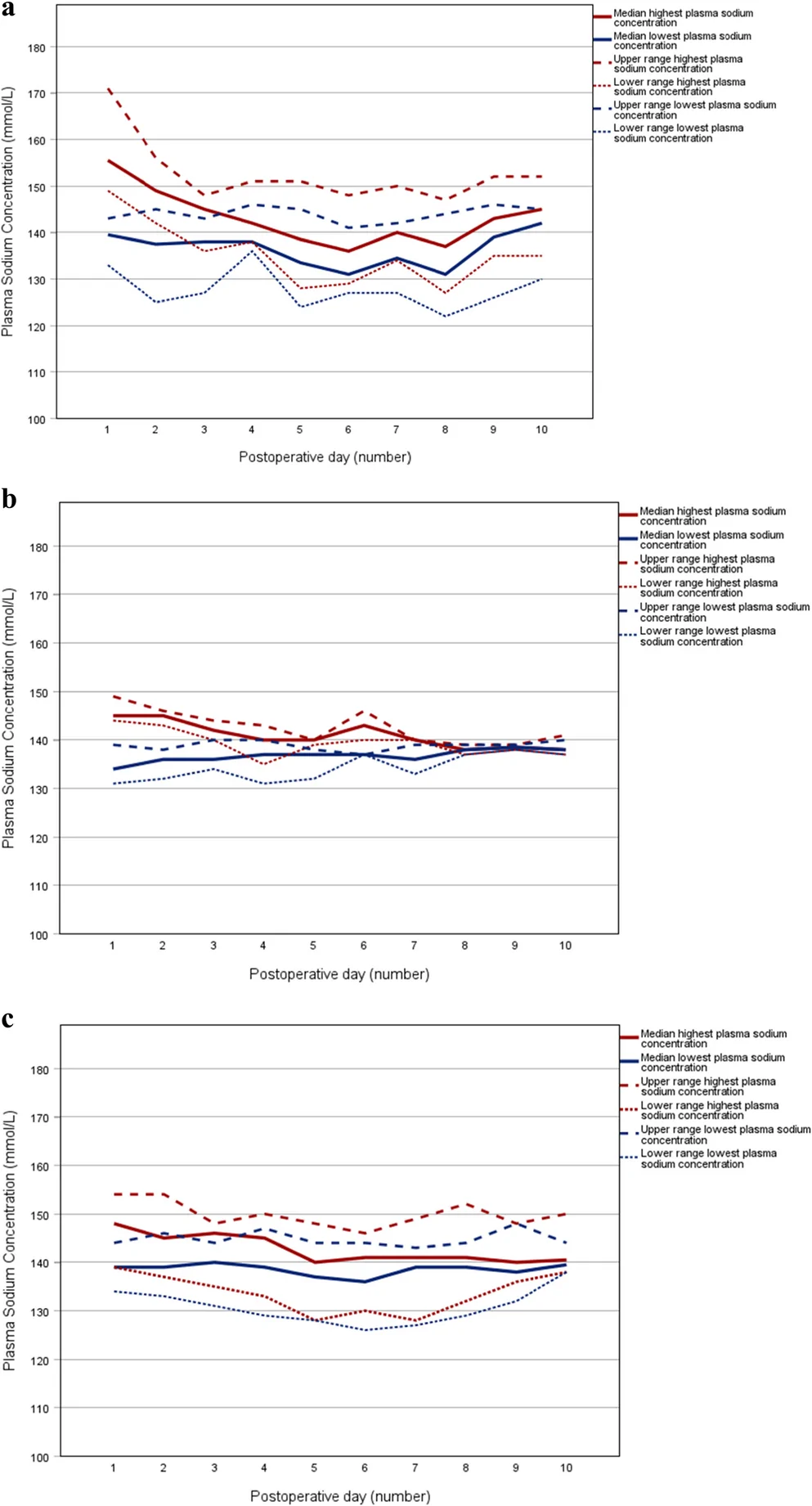

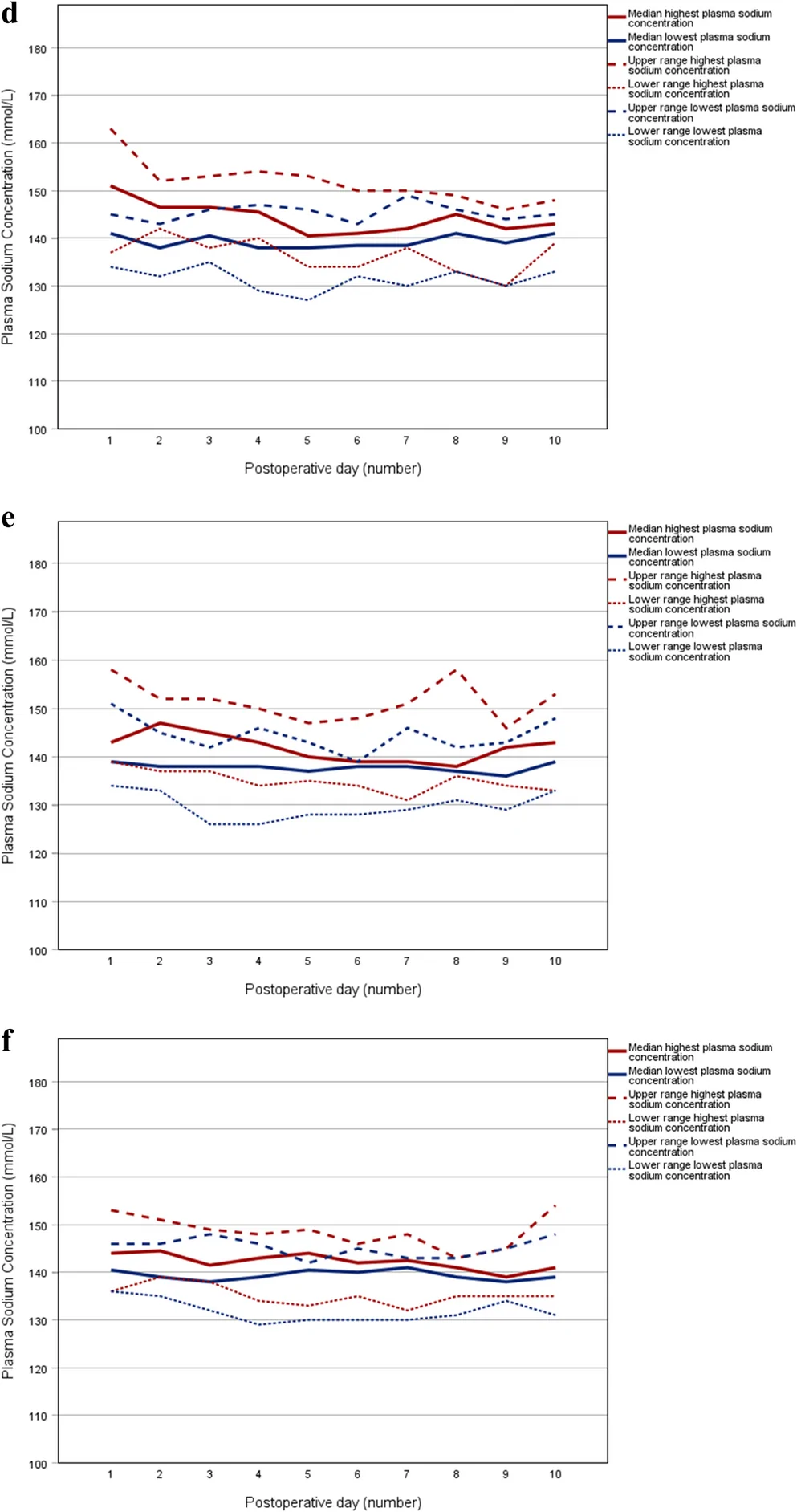
Median highest and median lowest plasma sodium concentration (mmol/L) per day (including upper and lower range) during the first 10 days after tumor surgery in patients with early postoperative AVP-D after centralization of care in the Netherlands, depicted per year. (**a**) 2018 (*n* = 6), (**b**) 2019 (*n* = 3), (**c**) 2020 (*n* = 13), (**d**) 2021 (*n* = 12), (**e**) 2022 (*n* = 9), (**f**) 2023 (*n* = 11)

In the first year since centralization of care, in 2018, the lowest and highest observed plasma sodium concentration during the first 10 days postoperatively ranged from 122 mmol/L to 171 mmol/L. In 2019, this range decreased to a minimal value of 131 mmol/L and a maximal value of 149 mmol/L; in 2020, from 126 mmol/L to 154 mmol/L; in 2021, from 127 mmol/L to 163 mmol/L; in 2022, from 126 mmol/L to 158 mmol/L; and in 2023, from 129 mmol/L to 143 mmol/L, respectively (Fig. 3a-f).

**Fig. 4:**
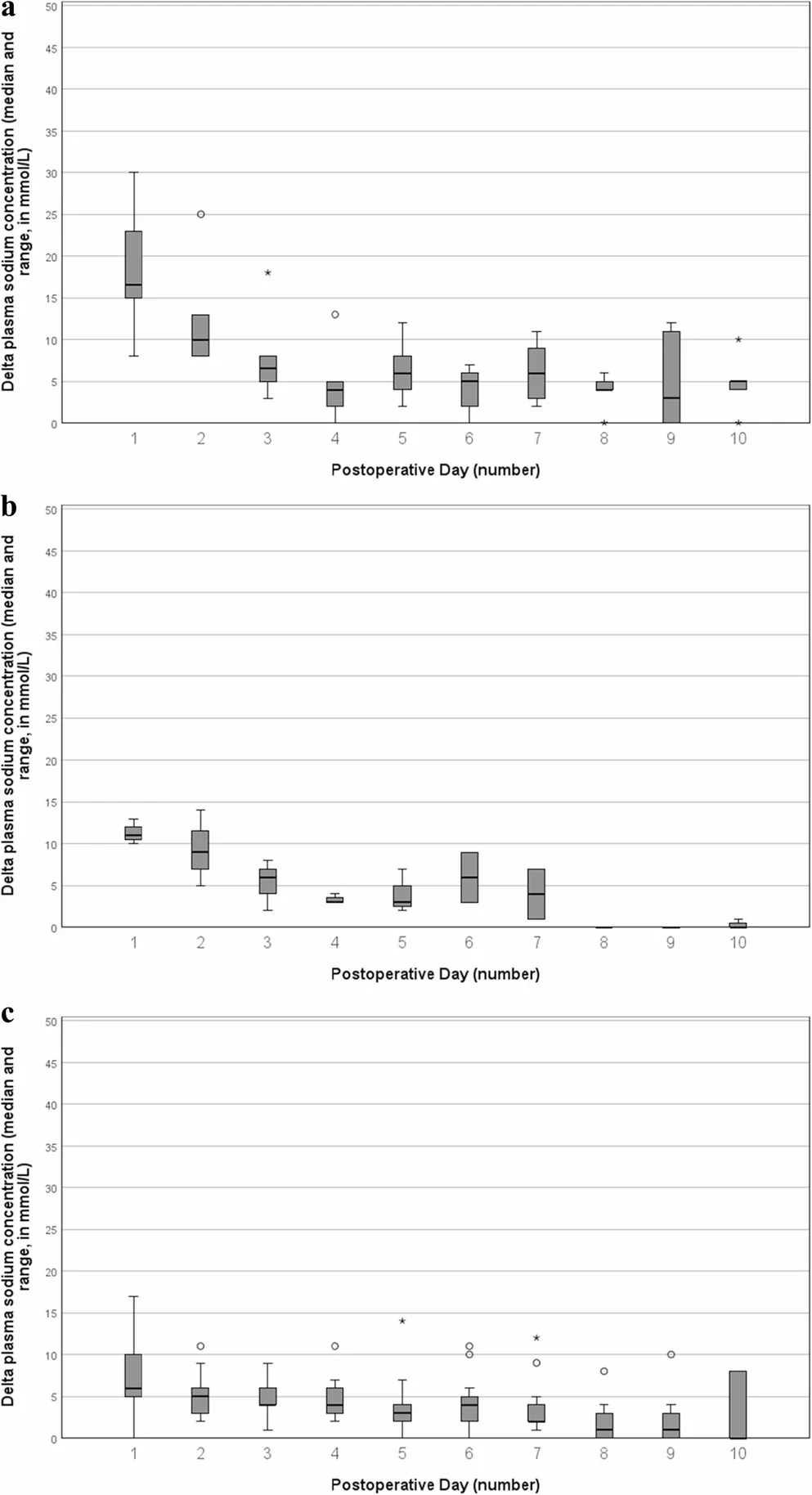

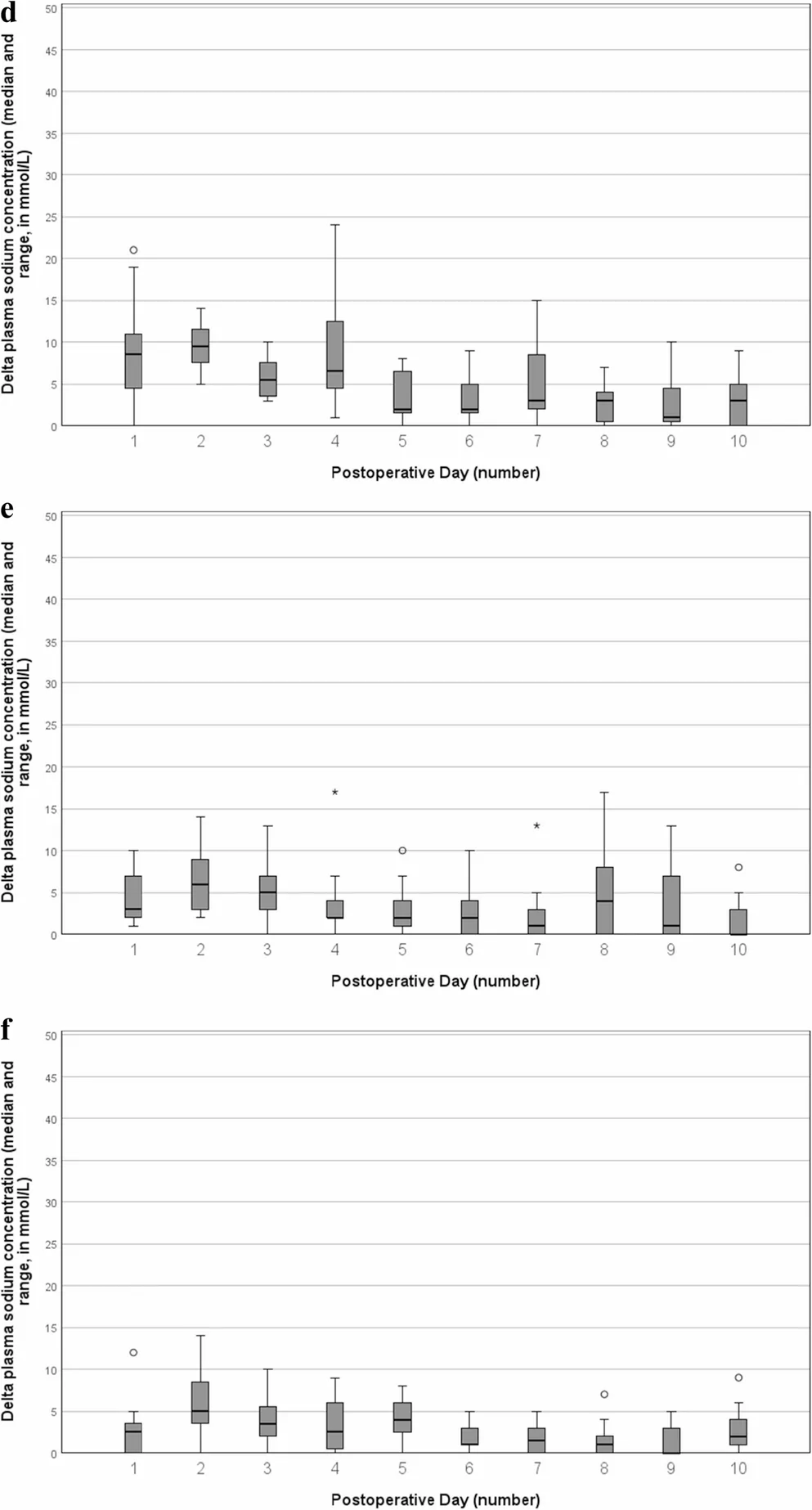
Delta plasma sodium concentration (mmol/L) per day (including median, upper and lower range) during the first 10 days after tumor surgery in patients with early postoperative AVP-D after centralization of care in the Netherlands, depicted per year. **a **2018 (n=6), **b** 2019 (n=3), **c** 2020 (n=13), **d** 2021 (n=12), **e** 2022 (n=9), **f** 2023 (n=11)

The highest observed delta plasma sodium concentration was 30 mmol/L in the cohort of 2018. In 2019, the highest delta plasma sodium concentration was 13 mmol/L; in 2020 17 mmol/L; in 2021 24 mmol/L; in 2022 17 mmol/L; and in 2023 14 mmol/L (Fig. 4a).

### Short- and long-term postoperative neurological events

The occurrence of short- and long-term neurological events in patients with and without early postoperative AVP-D is summarized in Table 2. Short-term neurological events included epileptic seizures, reported in 7/73 (9.6%) patients, and altered mental status, reported in 9/73 (12.3%) patients. Of the 7 epileptic seizures and 9 cases of altered mental state, 4/7 (57.1%) and 1/9 (11.1%) occurred during a hyponatremic episode. The occurrence of epileptic seizures did not differ significantly between patients with and without early postoperative AVP-D (*p* = 0.67), nor did the occurrence of altered mental status (*p* = 1.0). Of the long-term neurological events, visual impairment (45/73, 61.6%) and fatigue (26/73, 35.6%) were reported most frequently. None of the long-term postoperative neurological events differed significantly between patients with and without early postoperative AVP-D.

**Table 2:** Short-term (< 10 postoperative days) and long-term neurological consequences in all patients and in patients with or without early postoperative AVP-D in a univariate logistic regression model

	Total *n* (%)	Missing *n* (%)	Occurrence of early postoperative AVP-D yes *n* (%) no *n* (%)		*P* value	OR (95% CI)
	73	-	50 (68.5)	23 (31.5)	-	-
Short-term neurological events						
Epileptic seizure	7 (9.6)	-	4 (57.1)	3 (42.9)	0.671	0.602 (0.047–7.698)
Altered mental status	9 (12.3)	1	6 (66.7)	3 (33.3)	1.000	0.174 (0.023–1.352)
Long-term neurological events						
Headache	22 (30.1)	4	17 (77.3)	5 (22.7)	0.341	2.725 (0.605–12.280)
Epileptic seizure	4 (5.5)	-	2 (50.0)	2 (50.0)	0.586	1.441 (0.061–33.905)
Visual impairment	45 (61.6)	3	34 (75.6)	11 (24.4)	0.174	4.680 (1.119–19.566)
Motor deficit	10 (13.7)	-	6 (60.0)	4 (40.0)	0.715	0.796 (0.100–6.339.100.339)
Cognitive dysfunction	12 (16.4)	4	6 (50.0)	6 (50.0)	0.177	0.129 (0.021–0.779)
Sleep problems	8 (11.0)	1	6 (75.0)	2 (25.0)	1.000	1.472 (0.125–17.302)
Fatigue	26 (35.6)	2	20 (76.9)	6 (23.1)	0.202	2.337 (0.551–9.915)

*AVP-D* arginine vasopressin deficiency, *OR* odds ratio, *CI* confidence interval

### Presence of pituitary dysfunction at last moment of follow-up

At last moment of follow-up, after a median duration of 2.0 years (range 0.2–5.8), 27/73 patients (37.0%) had panhypopituitarism, and 72/73 (98.6%) had 2 or more hypothalamus-pituitary axes deficient. In 22 of 23 patients (95.7%) with a postoperative triphasic course, pituitary function was evaluated after three months (1 patient had deceased within 3 months). Of these 22, all (100.0%) had central hypothyroidism, of which 20 (91.0%) had both central hypothyroidism and ACTH deficiency, after three months. During follow-up, 12/22 patients (54.5%) had additionally been diagnosed with GH deficiency, and 7/22 (32.0%) with central hypogonadism.

## Discussion

In this retrospective cohort study we aimed to evaluate the postoperative management of plasma sodium fluctuations in children who underwent resection of sellar or suprasellar tumors since centralization of care at the Princess Máxima Center in the Netherlands. A similar evaluation was performed in a national cohort before the care for pediatric oncology patients had been centralized [26]. A first finding of this study is a decrease in both the delta sodium as well as in the minimum and maximal value of sodium was seen in the years following 2018. Since centralization of care, the most severe outliers were observed in 2018, which is the first year after centralization. In addition, the change in plasma sodium concentrations per 24 h in children with early postoperative AVP-D is lower than was reported in a comparable pediatric cohort of patients treated for (supra)sellar tumors in the Netherlands *before* centralization of care [26]. Furthermore, the absolute lowest and highest plasma sodium concentrations deviate less from the normal range, when compared to this previous cohort of patients with postoperative AVP-D. These findings seem to suggest an improved trend in the management of plasma sodium balance over the years since centralization in this vulnerable patient group, probably attributable to the improved expertise of all members of the multidisciplinary team after centralization of care and the collaborative implementation of a clinical protocol. Following centralization of care, the variability in plasma sodium concentration steadily decreased over successive years. Importantly, no causal relationship between centralization of care and improved sodium management can be established directly, since this study only described the cohort post-centralization. Also, we no longer found an association between early postoperative AVP-D and the occurrence of short-term neurological events, in contrast to findings from the national cohort before centralization [26]. This may partly be explained by our smaller sample size, which limits the ability to detect such associations. It must also be emphasized that these are results from a heterogenous group of (supra)sellar tumors. However, improved management of plasma sodium levels in patients with early postoperative AVP-D may also have played a role, as smaller fluctuations in sodium concentration reduce the risk of short-term neurological sequelae. Prior to centralization, protocols for clinical management of postoperative AVP-D were center-specific, and a limited number of patients per center were treated annually, potentially contributing to variation in recognition, management and clinical outcomes. With the care for pediatric (supra)sellar tumors now largely centralized across the Netherlands, all team members, from PICU nurses and ward physicians to the on-call pediatric endocrinologist, have developed expertise in the management of these patients with postoperative AVP-D.

The observed incidence of early postoperative AVP-D in our current cohort was 68.5%, which is similar to the previously reported prevalence of 67.5%, prior to centralization of care [26]. This emphasizes that AVP-D remains a prevalent and clinically relevant complication of pediatric (supra)sellar tumor resection. Furthermore, all patients with a postoperative triphasic response subsequently developed permanent AVP-D, and a triphasic response was observed in more than half of the patients with permanent AVP-D.

For management of the fluid balance in children at risk for AVP-D post-surgery, in our center a protocol has been developed in collaboration with the PICU, the department of endocrinology and pediatric oncology, which already starts preoperatively, and then continues peri-operatively and in the postoperative period (Supplement 1). In this protocol, the pediatric endocrinologist is the coordinating physician throughout all phases of care; from outpatient clinic to inpatient clinic, operating theater, PICU and back to inpatient clinic and dismissal to outpatient clinic, hereby providing continuation of care. In each phase, a different attending physician coordinates the plasma sodium concentration and fluid balance which may differ from attending resident to anesthesiologist, pediatric intensivist or pediatric neuro-oncologist. As the patient frequently changes from department within this treatment phase, it is crucial that there is a continuous coordinating physician that oversees the different stages of AVP-D development and desmopressin requirements. In our expert center, next to collaborative protocol writing, clinical lessons are provided to instruct and teach the team, that includes attending nurses, specialized nurses, (young) residents and supervising staff, about the pitfalls in AVP-D management. Pitfalls that should be recognized are the presence of adipsia (impaired thirst perception), peri-operative hypotension necessitating extra (intravenous) fluids, co-medication influencing diuresis or plasma sodium concentration (e.g. steroids or vincristine) and the presence of hypertonic dehydration (in the latter high sodium concentrations and concentrated urine are present, whereas, in AVP-D, high sodium concentrations with diluted urine are present). Severe shifts in plasma sodium concentration or rapid correction of hypo- or hypernatremia may affect neural integrity and may lead to acute neurological complications, such as cerebral edema following rapid correction of hypernatremia or central pontine myelinolysis resulting from overly rapid correction of hyponatremia [29, 30]; none of these complications were observed in the current study sample. Before centralization of care, a maximum, individually observed, delta plasma sodium concentration during the first ten postoperative days of patients with postoperative AVP-D of 46 mmol/L/24 h, was observed [26]. Although the decreased maximum delta here reported in 2018 (30 mmol/L/24 h) is still too high, it is shown that further improvement of this maximum delta could be observed over the subsequent years to a max of 14 mmol/L/24 h in 2023. These changes reflect an improvement in the management of changes in fluid and sodium balance postoperatively with prevention of too rapid or too steep changes in plasma sodium.

Despite centralization of care and improved expertise of the team, management of post-operative AVP-D remains challenging as can for example be seen in the outliers of serum sodium concentrations in 2021. These outliers were caused by a single case of a very young child (age 2 months) at time of surgery. In this specific case, the AVP-D was probably caused by severe shifts in intracranial pressure, rather than pituitary (stalk) damage. After 5 weeks, the AVP-D normalized and desmopressin could be stopped. This illustrates that in children with very young age (difficult to interpret feelings of thirst) or in case of unexpected AVP-D (with intact pituitary stalk and function) increased awareness is necessary to prevent severe changes in plasma sodium concentrations.

Several limitations of our study should be acknowledged. The retrospective nature of this study limits the ability to study the correlation between early postoperative AVP-D and the occurrence of short- and long-term neurological effects in depth, and has therefore been limited to an explorative analysis for associations. Additionally, plasma sodium concentrations are outcomes of point-of-care testing, as continuous monitoring of plasma sodium is not feasible, and may not always capture all fluctuations. Significant outliers could therefore potentially be missed. However, plasma sodium levels were measured routinely and frequently in each patient, following the local protocol, and were measured more frequently in response to abnormal values and changes in treatment, to ensure that potential extremes are either captured or prevented and will thus be mostly included in our data. We explored whether there was an association in this cohort between tumor type or pituitary stalk sacrifice and severe sodium fluctuations, but could not find such an association. A limitation of this exploration was the fact that not in all surgical reports it was explicitly stated whether the pituitary stalk was sacrificed or not. As expected, all patients with an explicitly stated sacrificed pituitary stalk, developed permanent AVP-D. The lack of association between tumor type and severe sodium fluctuations might be explained by the fact that postoperative AVP-D management is similar, regardless of tumor pathology. Also, the lack of association between noted pituitary stalk sacrifice in the surgical report and the severity of sodium fluctuations might be explained by the fact that more careful sodium/fluid monitoring is conducted by the team in these patients, given that postoperative AVP-D is anticipated when the neurosurgeon reports pituitary stalk transection. Moreover, due to the rareness of pediatric (supra)sellar brain tumors and AVP-D, the cohort size remains limited and lacks power for further statistical analyses. Although findings of this cohort were compared to the previous findings of the national cohort in the Netherlands prior to centralization of care, we did not perform statistical analyses for comparison. We reasoned it would not have been appropriate to use statistical tests for comparisons of outcomes of two different cohorts, since plasma sodium measurements have not been performed following similar protocols nor in similar labs in the years before centralization compared to after centralization of care. Furthermore, in our study, we chose to exclude patients who were already diagnosed with AVP-D (and thus already on maintenance desmopressin treatment) prior to tumor resection, since these patients are expected to have more stable plasma sodium levels postoperatively, as also reported by Kruis et al. [26]. Interestingly, even when only evaluating the postoperative course of plasma sodium levels of patients who are at highest risk of a complicated course (patients with newly diagnosed AVP-D), we see less severe plasma sodium fluctuations in the first 10 postoperative days. We believe this comparison might be considered a strength of this study, since this method allowed for a meaningful evaluation of care and provided clinically relevant insights into the effects of system-level changes in delivery of care for rare diseases.

The majority of patients with a postoperative triphasic response were diagnosed with at least hypothyroidism and ACTH deficiency at three months postoperatively. The presence of a triphasic response appears to be a valuable clinical indicator for the development of long-term endocrine dysfunction, although it is now based on descriptive data. In our experience, patients exhibiting this response are considerably more likely to experience multiple hormone deficiencies during follow-up, including permanent AVP-D.

In conclusion, findings of this study indicate that centralization of care for pediatric suprasellar brain tumors requiring neurosurgery may be associated with improved early postoperative management of sodium balance. This study enhances the awareness of the risks inherent to (supra)sellar tumor surgery and underlines the importance of centralization of care for rare diseases.

## Supplementary Information

Below is the link to the electronic supplementary material.

Supplementary Material 1 (DOCX 25.8 KB)

## Data Availability

The datasets generated and/or analyzed during the current study will be made available upon reasonable request. The request can be submitted to the corresponding author. All data have been anonymized to ensure patient privacy.

## References

[1] Lebbink CA et al (2021) Prevalence and risk factors of hypothalamic-pituitary dysfunction in infant and toddler childhood brain tumor survivors. Eur J Endocrinol 185(4):597–606 34324432 10.1530/EJE-21-0137

[2] van Roessel I et al (2025) The many different clinical faces of acquired hypothalamic dysfunction: a retrospective cohort study in the Netherlands. EClinicalMedicine 85:103313 40686687 10.1016/j.eclinm.2025.103313 PMC12270709

[3] Calandrelli R et al (2024) Pediatric craniopharyngiomas: magnetic resonance imaging assessment for hypothalamus-pituitary axis dysfunction and outcome prediction. Pediatr Radiol 54(1):157–169 38019284 10.1007/s00247-023-05814-3

[4] Calandrelli R et al (2024) Topography and Radiological Variables as Ancillary Parameters for Evaluating Tissue Adherence, Hypothalamic-Pituitary Dysfunction, and Recurrence in Craniopharyngioma: An Integrated Multidisciplinary Overview. Cancers (Basel), 16(14) 10.3390/cancers16142532 PMC11275213 39061172

[5] Arima H et al (2022) Changing the name of diabetes insipidus: a position statement of The Working Group for Renaming Diabetes Insipidus. Endocr J 69(11):1281–1284 36244744 10.1507/endocrj.EJ20220831

[6] Finken MJJ et al (2011) Frequent Occurrence of the Triphasic Response (Diabetes Insipidus/Hyponatremia/Diabetes Insipidus) after Surgery for Craniopharyngioma in Childhood. Hormone Res Paediatrics 76(1):22–26 10.1159/000324115 21701131

[7] Pratheesh R et al (2013) Incidence, predictors and early post-operative course of diabetes insipidus in paediatric craniopharygioma: a comparison with adults. Child’s Nerv Syst 29(6):941–949 23386174 10.1007/s00381-013-2041-8

[8] Williams C et al (2012) Hyponatremia with intracranial malignant tumor resection in children. J Neurosurgery: Pediatr PED 9(5):524–529 10.3171/2012.1.PEDS11465 PMC3358130 22546031

[9] Williams CN et al (2014) The incidence of postoperative hyponatremia and associated neurological sequelae in children with intracranial neoplasms: Clinical article. J Neurosurgery: Pediatr PED 13(3):283–290 10.3171/2013.12.PEDS13364 24410125

[10] Omar FA-Z, Bunyan MA (1997) Severe hyponatremia as poor prognostic factor in childhood neurologic diseases. J Neurol Sci 151(2):213–216 9349678 10.1016/s0022-510x(97)00133-0

[11] Tzoulis P, Bagkeris E, Bouloux P-M (2014) A case-control study of hyponatraemia as an independent risk factor for inpatient mortality. Clin Endocrinol 81(3):401–407 10.1111/cen.12429 24612060

[12] Williams CN et al (2015) Hyponatremia and poor cognitive outcome following pediatric brain tumor surgery. J Neurosurgery: Pediatr PED 15(5):480–487 10.3171/2014.10.PEDS14368 25723724

[13] Arieff AI, Llach F, Massry SG (1976) Neurological manifestations and morbidity of hyponatremia: correlation with brain water and electrolytes. Medicine 55(2):121–129 1256311 10.1097/00005792-197603000-00002

[14] Hannon MJ, Thompson CJ (2014) Neurosurgical hyponatremia. J Clin Med 3(4):1084–1104 26237593 10.3390/jcm3041084 PMC4470172

[15] Sterns RH, Riggs JE, Schochet SS Jr. (1986) Osmotic demyelination syndrome following correction of hyponatremia. N Engl J Med 314(24):1535–1542 3713747 10.1056/NEJM198606123142402

[16] Sterns RH et al (2024) Treatment Guidelines for Hyponatremia: Stay the Course. Clin J Am Soc Nephrology: CJASN 19(1):129–135 10.2215/CJN.0000000000000244 PMC10843202 37379081

[17] Atila C et al (2022) Central diabetes insipidus from a patient’s perspective: management, psychological co-morbidities, and renaming of the condition: results from an international web-based survey. Lancet Diabetes Endocrinol 10(10):700–709 36007536 10.1016/S2213-8587(22)00219-4

[18] SKION (SKN) (2025) ; Available from: https://www.skion.nl/

[19] Müller HL et al (2011) Post-operative hypothalamic lesions and obesity in childhood craniopharyngioma: results of the multinational prospective trial KRANIOPHARYNGEOM 2000 after 3-year follow-up. Eur J Endocrinol 165(1):17–24 21490122 10.1530/EJE-11-0158

[20] Samii M, Tatagiba M (1997) Surgical management of craniopharyngiomas: a review. Neurol Med Chir (Tokyo) 37(2):141–149 9059036 10.2176/nmc.37.141

[21] Bogusz A et al (2019) Posterior hypothalamus-sparing surgery improves outcome after childhood craniopharyngioma. Endocr Connect 8(5):481–492 30925462 10.1530/EC-19-0074 PMC6479199

[22] Sanford RA (1994) Craniopharyngioma: results of survey of the American Society of Pediatric Neurosurgery. Pediatr Neurosurg 21(Suppl 1):39–43 7841077 10.1159/000120860

[23] Madsen PJ et al (2019) Endoscopic endonasal resection versus open surgery for pediatric craniopharyngioma: comparison of outcomes and complications. J Neurosurg Pediatr 24(3):236–245 31174192 10.3171/2019.4.PEDS18612

[24] Madsen PJ et al (2022) Pediatric Pituitary Surgery. Otolaryngol Clin North Am 55(2):477–491 35256170 10.1016/j.otc.2021.12.017

[25] van Santen HM et al (2023) Diagnostic criteria for the hypothalamic syndrome in childhood. Eur J Endocrinol, 188(2) 10.1093/ejendo/lvad009 36737045

[26] Kruis RWJ et al (2018) Management and consequences of postoperative fluctuations in plasma sodium concentration after pediatric brain tumor surgery in the sellar region: a national cohort analysis. Pituitary 21(4):384–392 29623580 10.1007/s11102-018-0886-2 PMC6018586

[27] Cole TJ et al (2000) Establishing a standard definition for child overweight and obesity worldwide: international survey. BMJ 320(7244):1240–1243 10797032 10.1136/bmj.320.7244.1240 PMC27365

[28] Cole TJ et al (2007) Body mass index cut offs to define thinness in children and adolescents: international survey. BMJ 335(7612):194 17591624 10.1136/bmj.39238.399444.55 PMC1934447

[29] Sterns RH (2015) Disorders of plasma sodium–causes, consequences, and correction. N Engl J Med 372(1):55–65 25551526 10.1056/NEJMra1404489

[30] Liamis G et al (2006) *Therapeutic approach in patients with dysnatraemias.* Nephrology, dialysis, transplantation: official publication of the European Dialysis and Transplant Association. - Eur Ren Association 21(6):1564–1569 10.1093/ndt/gfk090 16449285

