# Correction to: The effect of centralized care on the management of postoperative fluctuations in plasma sodium concentration after pediatric suprasellar brain tumor surgery

**DOI:** 10.1007/s11102-026-01723-4

**Published:** 2026-08-23

**Authors:** S. C. Hulsmann, D. C. Zaal, J. P. J. van Gestel, M. Sie, O. H. J. Eelkman Rooda, K. M. van Baarsen, R. E. A. Musson, B. Bakker, E. E. S. Nieuwenhuis, E. W. Hoving, R. M. Wösten-van Asperen, H. M. van Santen

**Affiliations:** 1 Princess Máxima Center for Pediatric Oncology, Heidelberglaan 25, Utrecht, 3584 CS The Netherlands; 2 Department of Pediatric Endocrinology, Wilhelmina Children’s Hospital, University Medical Center Utrecht, Lundlaan 6, Utrecht, 3584 EA The Netherlands; 3 Department of Pediatric Intensive Care, Wilhelmina Children’s Hospital, University Medical Center Utrecht, Lundlaan 6, Utrecht, 3584 EA The Netherlands; 4 Department of Clinical Chemistry, University Medical Center Utrecht, Lundlaan 6, Utrecht, 3584 EA The Netherlands

**Correction: Pituitary (2026) 29:65**

**https://doi.org/10.1007/s11102-026-01666-w**

The authors wish to correct an error in Table 2 of the above-referenced article (https://doi.org/10.1007/s11102-026-01666-w), with regards to the odds ratio’s and 95% confidence intervals. The corrected table is provided below.

Table 2. Short-term (< 10 postoperative days) and long-term neurological consequences in all patients and in patients with or without early postoperative AVP-D in a univariate logistic regression model.

**Table:** 

	Total *n* (%)	Missing *n* (%)	Occurrence of early postoperative AVP-D yes *n* (%) no *n* (%)		*P* value	OR (95% CI)
Total patients	73	-	50 (68.5)	23 (31.5)	-	-
**Short-term neurological events**						
Epileptic seizure	7 (9.6)	-	4 (57.1)	3 (42.9)	0.671	0.580 (0.119–2.832)
Altered mental status	9 (12.3)	1 (1.4)	6 (66.7)	3 (33.3)	1.000	0.930 (0.211–4.103)
**Long-term neurological events**						
Headache	22 (30.1)	4 (5.5)	17 (77.3)	5 (22.7)	0.341	1.755 (0.547–5.628)
Epileptic seizure	4 (5.5)	-	2 (50.0)	2 (50.0)	0.586	0.438 (0.058–3.318)
Visual impairment	45 (61.6)	3 (4.1)	34 (75.6)	11 (24.4)	0.174	2.061 (0.721–5.888)
Motor deficit	10 (13.7)	-	6 (60.0)	4 (40.0)	0.715	0.648 (0.164–2.561)
Cognitive dysfunction	12 (16.4)	4 (5.5)	6 (50.0)	6 (50.0)	0.177	0.390 (0.110–1.390)
Sleep problems	8 (11.0)	1 (1.4)	6 (75.0)	2 (25.0)	1.000	1.465 (0.272–7.887)
Fatigue	26 (35.6)	2 (2.7)	20 (76.9)	6 (23.1)	0.202	2.024 (0.678–6.040)

AVP-D, arginine vasopressin deficiency; OR, odds ratio; CI, confidence interval

