# Supplementary material for: The effect of centralized care on the management of postoperative fluctuations in plasma sodium concentration after pediatric suprasellar brain tumor surgery
Source: Pituitary. 2026 Mar 29;29(2):65. doi: 10.1007/s11102-026-01666-w (PMC13033463; doi:10.1007/s11102-026-01666-w)
Supplement: Supplementary file 1 — Supplementary Material 1 (DOCX 25.8 KB) [file 11102_2026_1666_MOESM1_ESM.docx]

**Protocol for Fluid Management in the Pediatric Intensive Care Unit for Children at risk of AVP-deficiency or SIADH**

**Subject**

Pediatric Care Protocol
Treatment of children in the intensive care unit (ICU) with (risk of) arginine vasopressin deficiency (AVP-D). This often concerns children immediately post-operative following the removal of a craniopharyngioma or another (supra)sellar tumor (e.g., germinoma, prolactinoma, ACTH-producing adenoma, pituitary macroadenoma).

**Objective**

This protocol outlines the treatment of AVP-D in children, with a primary focus on fluid and electrolyte balance. This management can be complex, especially in the immediate postoperative period after tumor removal in the sellar or suprasellar region, where phases of AVP-D may alternate with phases of **SIADH** (Syndrome of Inappropriate Antidiuretic Hormone Secretion).

**Scope of Practice**

The management of AVP-D in pediatric ICU patients is a medical procedure and must be performed under supervision of physicians.

**Target Audience**

Physicians, nurses, and physician assistants working in the pediatric intensive care unit or wards where these patients are admitted.

**Protocol Content**

**PREOPERATIVE PHASE**

**For children scheduled for resection of a craniopharyngioma or other suprasellar tumor.**

**Assessments and tests:**

- Complete physical examination including height, weight, and pubertal staging.
- Medical history focusing on drinking and urination patterns (often patients and parents are unaware of polyuria).
- Growth charts should be reconstructed as fully as possible, including parental measurements.

**Laboratory investigations:**

1. Complete blood count (CBC) with leukocyte differential and platelet count
2. Urea, creatinine, liver enzyme (aspartate aminotransferase; AST), albumin
3. Activated partial thromboplastin time (APTT), prothrombin time (PT), crossmatch serum, blood type, Rhesus factor
4. Sodium, potassium, chloride, osmolality, blood gas, glucose
5. Insulin-like growth factor 1 (IGF-1), insulin-like growth factor binding protein 3 (IGFBP-3), free T4, thyroid stimulating hormone (TSH), prolactin, morning cortisol, adrenocorticotropic hormone (ACTH) (if not yet on steroids)
6. For children >11 years: luteinizing hormone (LH), follicle-stimulating hormone (FSH), testosterone/estradiol; further evaluation as needed in consultation with endocrinologist
7. Urinalysis with dipstick and sediment
8. Tumor markers: alpha-fetoprotein (AFP), human chorionic gonadotropin (HCG)

**Clinical preparations:**

- Begin 24-hour fluid balance charts and daily weight measurement if possible.
- If the child is known to have hypocortisolism, start hydrocortisone stress dosing.

**INTRAOPERATIVE MANAGEMENT**

**1. Fluid Management:**

- For patients already using Desmopressin (Minrin/DDAVP), the endocrinologist determines the preoperative dose.
- Note that even children with pre-existing AVP-D may go through a postoperative SIADH phase.
- **AVP-D indicators**: High urine output, rising/high plasma sodium, urine osmolality < 100 mOsm/kg
- **SIADH indicators**: Positive fluid balance, low/decreasing serum sodium, urine osmolality > 100 mOsm/kg without glucosuria
- The anesthesiologist manages intraoperative fluids (preferably start with insensible loss infusion at 300 ml/m²/day and separate diuresis infusion).

**2. Hypocortisolism:**

- Children known to have adrenal insufficiency should begin hydrocortisone stress dosing the night before surgery unless dexamethasone has been started by neurosurgery.
- All children undergoing surgery in the pituitary region should receive peri-operative hydrocortisone stress dosing unless adequately covered by dexamethasone.

**POSTOPERATIVE MANAGEMENT**

Many children experience a **triphasic diuresis pattern**:
**AVP-D – SIADH – AVP-D**, corresponding risks of rapid sodium shifts. Therefore, they remain in the ICU for at least **72 hours** postoperatively for hourly urine monitoring.

If no signs of AVP-D or SIADH are observed in the first 48 hours, early transfer to the ward may be discussed with the pediatric endocrinologist. A **warm handover** is essential. The endocrinologist must be informed of the transfer.

**Upon ICU admission:**

- Assess hydration status (blood pressure (BP), central venous pressure (CVP), capillary refill, peripheral pulses, heart rate, weight, temperature changes).
- Correct fluid overload or deficit within 24 hours.

**First 48 Hours Postoperative**

**Intravenous (IV) fluids:**

1. Most patients return from the operating room fluid-overloaded. The balance can be slightly negative in the first 48 hours.
   - **No standard maintenance fluid** is started; only a **variable diuresis infusion** is given.
   - **Check serum potassium regularly** (as potassium is not supplemented routinely).
2. **Variable diuresis infusion**:
   - Match urine output hourly with **Glucose 2.5%- Sodium Chloride 0.45% infusion**, either IV or orally.
   - Ensure adequate glucose intake, especially in infants.

**Desmopressin (DDAVP):**

- Delay administration until **urine output >100 ml/m²/hour for 2 hours**, and serum sodium is stable/high or rising.
- If increased diuresis occurs, send blood and urine for **serum and urine osmolality + sodium**.
  - **AVP-D diagnosis**: Serum osmolality > 300 mOsm/kg with urine osmolality < 100 mOsm/kg
  - Urine sodium will be very low in AVP-D
  - Check for glucosuria
- **If DDAVP is indicated**:
- **IV dosing**:
  - IV: 0.1 mcg (under 1 year), 0.2 mcg (>1 year).
  - If no effect within 30 minutes, repeat dose.
  - Wait for urine output >100 ml/m²/hour and rising serum sodium/osmolality before next dose.

**Oral DDAVP Dosing**

- <2 years: 10 mcg
- 2–8 years: 25 mcg
- 8–14 years: 25–50 mcg
- 14 years: 50 mcg
  - IV Minrin can also be given orally if oral capsules are unavailable.

**Lab Monitoring (normal course):**

- Serum sodium, potassium, glucose: every 2 hours for 4 hours, then every 4 hours
- Hemoglobin (Hb), hematocrit (Hct), blood gas: every 12 hours
- Urea, creatinine: every 24 hours
- Urine osmolality: every 2 hours

**Clinical Monitoring:**

- CVP, BP, HR, RR, consciousness, pupils, temperature: hourly
- Urine output and fluid balance: hourly

**48–72 Hours Postoperative**

**IV Fluids:**

1. If well hydrated, start **fixed maintenance fluids**:
   - 300 ml/m²/24h NaCl 0.9% + 1–2 mmol KCl/kg/24h
2. Add variable diuresis fluids hourly:
   - Urine output + insensible loss replaced with Glucose 2.5%-NaCl 0.45%
   - Can be oral if tolerated.
   - Watch for SIADH as part of triphasic DI course.
   - Ensure adequate glucose intake in infants.

**Lab Monitoring:**

- Serum Na: 2x/day or before each Desmopressin dose
- Urine osmolality and sodium: every 4 hours or before Desmopressin

**Clinical Monitoring:**

- CVP, BP, HR, respiratory rate (RR): hourly
- Urine output and fluid balance: hourly
- Pupils, Glasgow Coma Scale (GCS), temperature: as clinically indicated and in consultation with neurosurgery
- Daily weights

**After 72 Hours Postoperative**

**Fluid Management:**

- Reassess volume status (weight, HR, capillary refill, serum sodium, osmolality).
- If **euvolemic**, reduce frequency of fluid replacement to **every 3 hours**.
- Encourage **free oral intake** based on thirst (note: not always reliable).
- Do **not replace** the last hour’s diuresis before this reassessment

**Lab Monitoring:**

- Serum sodium, potassium, glucose: 2x/day or before each DDAVP dose
- Urine sodium, potassium, osmolality: 4x/day or before DDAVP

**Clinical Monitoring:**

- BP, HR, RR: every 8 hours (or more if needed)
- Pupils, GCS, temperature: as clinically indicated
- Urine output and fluid balance: every 3 hours

**Additional Considerations During Postoperative Period**

- Compensate any external drain losses fully with sodium chloride 0.9% infusion every 6 hours
- Hyperglycemia may occur due to dexamethasone; treat with Novorapid if glucose >15 mmol/L for >2 hours
- Maintain glucose concentration between 6–10 mmol/L

**Hydrocortisone Stress Dosing:**

- Continue for 24 hours post-op (first 24h as prednisolone per protocol)
- If clinically stable and no indication for dexamethasone, stop hydrocortisone on day 2 to evaluate hypothalamic pituitary adrenal (HPA) axis
- Measure morning cortisol on days 3 and 4
- Restart stress dosing if hypocortisolism is suspected or during stress/fever

**Transfer from ICU to Ward**

- Only transfer when fluid and electrolyte balance is stable
- Confirm whether hourly urine monitoring is feasible on the ward
- Provide **warm handover** and inform pediatric endocrinologist
- **Urinary catheter** remains in place unless agreed otherwise
- Provide a **printed fluid balance summary** from ICU

**Note:**

- **Thyroid and growth hormone testing is not reliable in the first postoperative week**
- Check **FT4 after 1 week**
